# Effects of fecal microbiota transplantation on the abundance and diversity of selected fungal and archaeal species in the gut microbiota in the rat model of schizophrenia

**DOI:** 10.1007/s43440-025-00793-8

**Published:** 2025-10-22

**Authors:** Agnieszka Krawczyk, Tomasz Kasperski, Tomasz Gosiewski, Agnieszka Nikiforuk, Agnieszka Potasiewicz, Zbigniew Arent, Dominika Salamon

**Affiliations:** 1 Department of Molecular Medical Microbiology, Jagiellonian University Medical College, Kraków, Poland; 2 Chair of Microbiology, Jagiellonian University Medical College, Krakow, Poland; 3 Microbiome Research Laboratory, Department of Molecular Medical Microbiology, Jagiellonian University Medical College, Kraków, Poland; 4 Department of Behavioural Neuroscience and Drug Development, Polish Academy of Sciences, Maj Institute of Pharmacology, Kraków, Poland; 5 Department of Infectious Diseases and Public Health, Faculty of Veterinary Medicine, University of Agriculture in Kraków, Kraków, Poland

**Keywords:** Fecal microbiota transplantation, Schizophrenia, Gut microbiota, Fungi, Archaea

## Abstract

**Background:**

The gut microbiome has been increasingly recognized for its potential role in schizophrenia through gut-brain interactions involving immune, neural, and metabolic pathways. This pilot study evaluated the impact of fecal microbiota transplantation (FMT) on the abundance and variability of selected fungal and archaeal species in the gut microbiota in the rat model of schizophrenia.

**Methods:**

Sprague-Dawley rats using as a prenatal methylazoxymethanol acetate (MAM-E17) model of schizophrenia underwent FMT or placebo. Fecal DNA was extracted and analyzed via quantitative Real-Time PCR (qPCR) to quantify selected fungi *(Candida tropicalis*, *Malassezia* spp., *Cryptococcus neoformans*) and archaea (*Methanobrevibacter smithii*, *Methanosphaera stadtmanae*) before and after intervention

**Results:**

A slightly higher prevalence of *C. tropicalis* was noted in MAM-exposed rats compared to healthy controls (19% vs. 10%). Post-FMT, *C. tropicalis* colonization increased to nearly 100% across all groups, irrespective of transplantation source, indicating natural microbiome maturation rather than FMT effect. *Malassezia* spp. were commonly present before treatment, with their abundance significantly declining after both FMT and placebo administration, suggesting procedural impacts rather than specific FMT effects. *C. neoformans* and methanogenic archaea were absent.

**Conclusions:**

Overall, the results suggest that FMT has limited impact on gut fungal populations, possibly due to the developmental stage of microbiome maturation or procedural interventions. The absence of archaea underscores the complexity of the microbiome’s role in neurodevelopmental disorders, highlighting the necessity for continued research into microbial influences on schizophrenia pathophysiology.

## Graphical Abstract

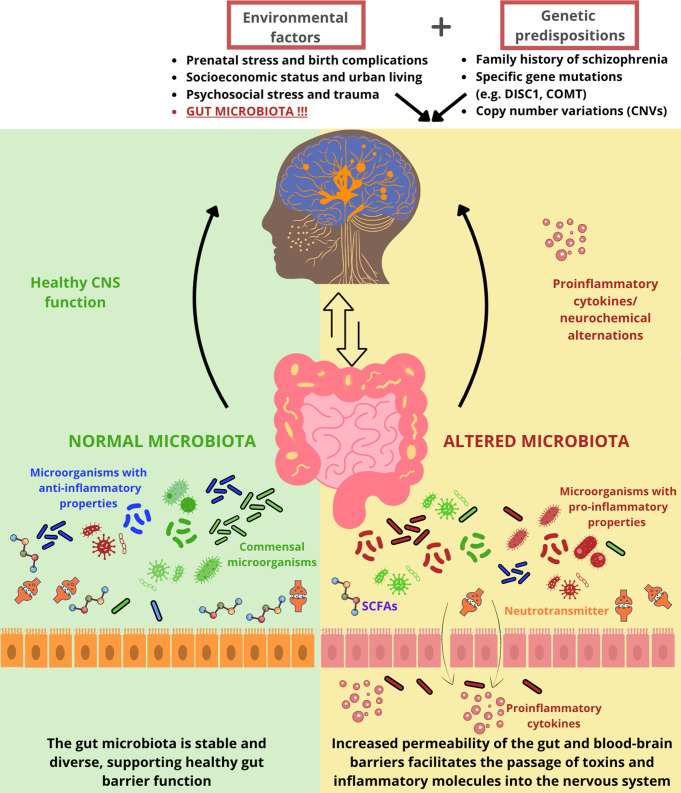

## Introduction

Schizophrenia is a complex psychiatric disorder characterized by a range of symptoms, including hallucinations, delusions, cognitive impairments or inappropriate affect [1].

Although the etiology of schizophrenia remains unclear, research indicates complex interaction that involves genetic, environmental, and neurological factors [2]. Schizophrenia has a strong genetic basis, with heritability estimated at approximately 80%. Recent genome-wide association studies (GWAS) have identified multiple genetic loci associated with the disorder, including genes involved in synaptic function, immune response, and calcium signaling [3]. Additionally, emerging evidence indicates that environmental factors, such as maternal infection, childhood trauma, cannabis use, urban living, and migration may influence or modify the impact of genetic predisposition. [4–6].

Recent research has increasingly emphasized the role of the gut-brain axis, particularly the gut microbiome, in the development and progression of schizophrenia [7]. This bidirectional communication occurs through multiple pathways, including neural circuits such as the vagus nerve, hormonal signaling via the hypothalamic-pituitary-adrenal (HPA) axis, and immune as well as metabolic mechanisms [7]. Imbalances in the gut microbiota, known as dysbiosis, may disrupt these pathways and contribute to the pathophysiology of schizophrenia [7]. For example, increased intestinal permeability (commonly referred to as „leaky gut”) allows bacterial toxins to enter the bloodstream, triggering systemic inflammation that can affect brain function and exacerbate psychiatric symptoms [8]. Moreover, the gut microbiome plays a crucial role in tryptophan metabolism, an amino acid precursor to serotonin, which has been implicated in schizophrenia [9]. Alterations in tryptophan processing can lead to the production of metabolites that influence glutamatergic neurotransmission, potentially contributing to cognitive deficits and negative symptoms observed in this disorder [9].

The kynurenine pathway of tryptophan metabolism is particularly important and has emerged as a key link between the gut microbiome and brain function in schizophrenia. Under physiological conditions, tryptophan is primarily metabolized via the kynurenine pathway, initiated by the enzymes indoleamine 2,3-dioxygenase (IDO) and tryptophan 2,3-dioxygenase (TDO). This process produces kynurenine, which can be further metabolized into various neuroactive compounds. One pathway leads to the formation of kynurenic acid, a metabolite with neuroprotective properties due to its antagonistic effects on N-methyl-D-aspartate receptors (NMDARs) and α7 nicotinic acetylcholine receptors (α7nAChR) [10]. The second pathway involves the production of quinolinic acid, a potent NMDAR agonist with excitotoxic and proinflammatory properties. Growing evidence suggests that disturbances in the kynurenine pathway may contribute to cognitive deficits, negative symptoms, and particularly treatment-resistant features of schizophrenia [11]. Notably,, altered kynurenine metabolism has been linked to treatment-resistant schizophrenia, where excessive modulation of NMDAR function appears to be the cause of persistent symptom dimensions [11]. Furthermore, gut microbiota may influence this pathway by regulating host immune activation and tryptophan availability, representing a biological mechanism through which dysbiosis can affect brain function in schizophrenia [12].

Dysbiosis has been implicated in various neuropsychiatric disorders, including schizophrenia [13]. Specific microbial species have been identified that can produce neurotransmitters or their precursors, potentially impacting central nervous system function [14, 15] and clinical studies have reported significant differences in the composition of the bacteriobiome between individuals with schizophrenia and healthy controls [16–20]. Fungal and archaeal components of the gut microbiome have also gained increasing attention in schizophrenia research. Recent studies show altered fungal diversity, including elevated levels of *Candida* species, which may contribute to immune dysregulation observed in patients with schizophrenia. Although research on archaea is still relatively limited, studies have reported an increased abundance of methanogenic species such as *Methanobrevibacter smithii* [21, 22], which may play a role in modulating gut–brain axis signaling and metabolic homeostasis in affected individuals. [23] Interestingly, animal models have demonstrated that fecal microbiota transplantation (FMT) from schizophrenia patients into germ-free mice can induce behavioral and neurochemical changes relevant to schizophrenia, suggesting a causal relationship between gut microbiome alterations and the disorder [24].

In view of the above and considering the growing evidence on the microbiome’s involvement in the pathophysiology of schizophrenia, the aim of our study was to determine the presence and abundance of selected microorganisms (specific species of fungi, and rarely analyzed archaea) in the rats before and after FMT, using quantitative Real-Time PCR (qPCR). While most microbiome studies in schizophrenia have focused on bacteria, fungi and archaea remain underexplored despite their potential relevance to host immunity, metabolism, and gut-brain interactions. We therefore concentrated on these microorganisms to complement existing bacteriome-focused research.

## Materials and methods

### Animal subjects

The study involved a total of 88 Sprague-Dawley rats, divided into two main groups: 48 prenatally MAM-exposed rats (a neurodevelopmental model of schizophrenia) and 39 healthy controls (HC). The prenatal methylazoxymethanol acetate (MAM-E17) rodent model is a well-established neurodevelopmental model that exhibits various histological, neurophysiological, and behavioral abnormalities similar to those observed in individuals with schizophrenia [25]. Among the schizophrenia-like rats, 26 were male and 22 were female. The control group consisted of 19 male and 20 female rats. All rats were raised under standard laboratory conditions and provided with food and water. The care and use of animals were in accordance with institutional guidelines for ethical animal research (approval of the 2nd Local Institutional Animal Care and Use Committee in Krakow, issued on 21 July 2022, resolution number 213/2022).

Each main group (MAM and HC) was further subdivided based on the treatment they received, resulting in four experimental groups:

- MAM + placebo (*n* = 22): prenatally MAM-exposed rats receiving a physiological saline solution
- MAM + FMT (*n* = 24): prenatally MAM-exposed rats receiving fecal suspension from healthy controls.
- HC + placebo (*n* = 19): healthy control rats receiving a physiological saline solution - NaCl 0.9%)
- HC+FMT (*n* = 19): healthy control rats receiving fecal suspension from MAM-exposed rats.

For FMT, fresh fecal samples were collected and prepared without freezing, and the suspensions were administered to the rats via gavage.

Fecal samples for DNA extraction were collected from both the MAM group and the HC group 30 days after the end of the triple FMT experiment, i.e. on the 86th day of life of the rats. and immediately frozen at −80 °C for later analysis.

### Preparation and administration of fecal suspension for fecal microbiota transplantation

A procedure designed in accordance with the recommendations provided by Gheorghe et al. [26] and Bokoliya et al. [27] was used to prepare the fecal material for transplantation. Immediately after collection, the feces were mixed with sterile saline solution (Chempur, Piekary Śląskie, Poland, NaCl 0.9%, product number: 117941206) to obtain a homogeneous fecal suspension. The mixture was then thoroughly homogenized and passed through a sterile nylon cell strainer with a pore size of 70 µm (Googlab, Rokocin, Poland, product number: SKU: 15–1070) to remove large particulate matter and obtain a smooth suspension. The prepared solution was then aliquoted and stored at −80 °C until use. Importantly, as previously reported by Bokoliya et al. [27] storage at −80 °C for up to six months does not significantly alter the microbial composition of fecal material intended for FMT.

**Fig. 1:**
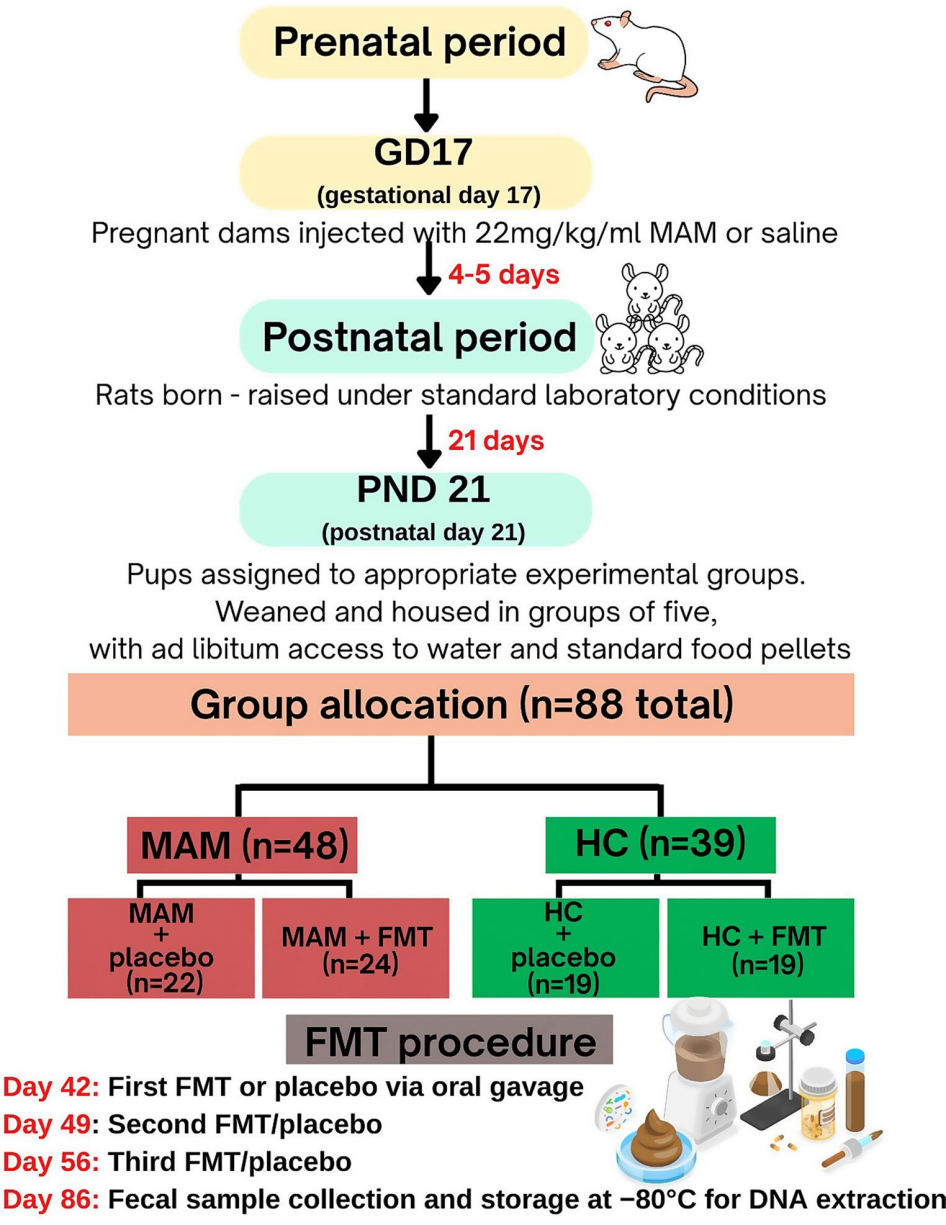
Schematic representation of the study design. FMT - fecal microbiota transplantation; GD17 -17th gestation day; HC - healthy control; MAM - methylazoxymethanol acetate; PND21–21st postnatal day

On day 42 of life, each rat received 500 µl of the fecal suspension via oral gavage using a gastric tube. The administered volume was calculated based on the average body weight of the rats, which were of similar size. The FMT procedure was performed three times at seven-day intervals, with subsequent doses given on day 49 and day 56 of life. Rats in the placebo groups received the same volume of sterile saline following the same schedule. After completing a full cycle of FMT, fecal samples were collected from individual rats belonging to each experimental group for DNA extraction and subsequent qPCR analysis, as described in the following section. Fig. 1 shows a flowchart summarizing the experimental design, schedule, and procedures performed.

### DNA extraction from stool samples

Stool samples were thawed, and 150 mg of each was weighed to allow for later calculation of microbial cell numbers per gram of feces. DNA from fungi and archaea was extracted using the high-efficiency Genomic Mini AX Stool kit (A&A Biotechnology, Gdansk, Poland, product number: 065–60), using our previously described modified protocol [28]. Briefly, archaeal and fungal cells were first lysed using lysozyme (Sigma-Aldrich, Poznań, Poland; 1 mg/ml, product number: L6876) and lysostaphin (A&A Biotechnology, Gdańsk, Poland; 3000 U, product number: 1007–3), followed by incubation of the samples at 37 °C for 30 min. Subsequently, 200 μl of 75 mM NaOH (Avantor, Gliwice, Poland, product number 1310–73-2) was added, and the mixture was heated at 95 °C for 10 min. Following incubation, samples were centrifuged at 12 000 rpm for 10 min, the supernatants discarded, and the pellets resuspended in 500 μl of buffer containing 2-mercaptoethanol (Sigma-Aldrich, Poznań, Poland, product number: 60–24-2). Lyticase (A&A Biotechnology, Gdansk, Poland; 10000 U, product number: 1018–10) was then added to each sample, which were incubated at 37 °C for 30 min and centrifuged again at 12 000 rpm for 10 min. DNA extraction was completed according to the protocol provided by A&A Biotechnology (Gdansk, Poland, product number: 065–60).

DNA yield and quality were assessed using a NanoDrop spectrophotometer (Thermo Fisher Scientific, Waltham, MA, USA), measuring absorbance at 260 nm and determining the 260/280 nm ratio to evaluate concentration and purity.

### Quantitative real-time PCR (qPCR)

The DNA obtained from the extraction process was used to detect specific fungal species (*Malassezia* spp., *Candida tropicalis*, and *Cryptococcus neoformans*) and two methanogenic archaea (*Methanobrevibacter stadtmanae* and *Methanosphaera smithii*). Before FMT, the presence and abundance of selected microorganisms were assessed in all fecal samples, and qPCR analysis was repeated on new stool samples collected after the third FMT dose, at the 30-day follow-up. Detection was carried out using TaqMan probes labeled with the FAM fluorescent dye (Genomed, Warsaw, Poland). Each PCR reaction consisted of: 2 μl of DNA template, 5 μl of qPCR HS Mix Probe (A&A Biotechnology, Gdansk, Poland, product number: 2008HS-100P), 0.2 μl of each primer (20 mM, Genomed, Warsaw, Poland), 0.6 μl of the TaqMan probe (10 mM, Genomed), and 2 μl of nuclease-free molecular biology grade water (A&A Biotechnology, Gdansk, Poland, product number: 005–2515). Primer and probe sequences [29–32] are listed in Table 1. The reactions were performed in optical strips designed for the CFX96 thermal cycler (BioRad, California, USA, product number: TCS0803EDU). Positive and negative controls were included in each run. Negative controls involved water (A&A Biotechnology, Gdansk, Poland, product number 005–2515) instead of DNA. Positive controls included genomic DNA obtained from cultured reference strains of fungi (*M. furfur* ATCC 14521, *C. tropicalis* ATCC 13803, and *C. neoformans* ATCC 204092), from which DNA was isolated in our laboratory. For archaeal targets (*M. smithii* DSM 861 and *M. stadtmanae* DSM 3091), purified genomic DNA from reference collections was used directly. The thermal conditions of the reaction are presented in Table 1. Amplification was measured at the cycle where the fluorescence signal crossed the cycle threshold (Ct). Standard curves were created using 10-fold serial dilutions (10^1^ to 10^7^ CFU/ml) of reference fungal strains and archaeal DNA. These curves allowed for the quantification of microbial load in positive samples based on their Ct values.

**Table 1:** Primer and probe sequences used for qPCR detection of selected fungal and archaeal species (*C. tropicalis*, *Malassezia* spp., *C. neoformans*, *M. smithii*, *M. stadtmanae*), along with the qPCR thermal cycling conditions

Microorganism/Sequence (5’ to 3’)	Thermal cycling programme
*C. tropicalis* [29] forward primer :GCGGTAGGAGAATTGCGTT reverse primer: TCATTATGCCAACATCCTAGGTTTA probe: 6-FAM-CGCAGTCCTCAGTCTAGGCTGGCAG-BHQ-1	2 min at 50 °C 10 min at 95 °C followed by 40 cycles of: 15 s at 95 °C 1 min at 60 °C
*C. neoformans* [30] forward primer: GCCGCGACCTGCAAAG reverse primer: GGTAATCACCTTCCCACTAACACAT probe: 6-FAM-ACGTCGGCTCGCC-BHQ-1	2 min at 50 °C 10 min at 95 °C followed by 40 cycles of: 15 s at 95 °C 1 min at 60 °C
*Malassezia spp.* [31] forward primer: GTAGACTCCATCTAAAGCTAAAT reverse primer: CTTTTAACTCTCTTTCCAAAGT probe: 6-FAM-CCCTCACGGTACTTGTTCGCT-BHQ-1	2 min at 94 °C followed by 40 cycles of: 20 s at 94 °C 20 s at 50 °C 30 s at 60 °C
*M. smithii* [32] forward primer: CCGGGTATCTAATCCGGTTC reverse primer: CTCCCAGGGTAGAGGTGAAA probe: 6- FAM-CCGTCAGAATCGTTCCAGTCAG- BHQ-1	15 min at 95 °C followed by 45 cycles of: 30 s at 95 °C 1 min at 60 °C
*M. stadtmanae* [32] forward primer:AGGAGCGACAGCAGAATGAT reverse primer: CAGGACGCTTCACAGTACGA probe: 6- FAM-TGAGAGGAGGTGCATGGCCG- BHQ-1	15 min at 95 °C followed by 45 cycles of: 30 s at 95 °C 1 min at 60 °C

### Statystical analysis

To compare the CFU values between groups non-parametric Kruskal-Wallis test with post hoc Dunn’s test with Bonferroni correction was performed. The prevalence of microorganisms between groups was analyzed using the Chi-square test or Fisher’s exact test, depending on group sizes. A p-value less than 0.05 was considered statistically significant.

## Results

A DNA purity of ≥1.7 was assumed. All samples examined met this criterion.

Before FMT, *C. tropicalis* was detected in 9 out of 48 rats (19%) in the MAM-exposed group and in 4 out of 39 (10%) healthy controls. There was no statistically significant difference in the prevalence between these two groups (χ^2^ = 1.22, df = 1, *p* = 0.212). In MAM + placebo group, *C. tropicalis* was detected in 21 out of 22 rats (95%). In the group MAM + FMT, *C. tropicalis* was detected in all 24 rats (no statistically significant difference was found between these group: (χ^2^ = 1.16, df = 1, *p* = 0.468). Among rats from group HC + FMT, *C. tropicalis* was detected in all 19 individuals. Similarly, *C. tropicalis* was also found in all 19 HC + placebo.

Before FMT, *Malassezia* was detected in 48 out of 48 rats (100%) in the MAM-exposed group and in 39 out of 39 (100%) healthy controls. In the MAM + placebo group, *Malassezia* was detected in all 22 rats (100%), and in 23 out of 24 rats (96%) from MAM + FMT group. Among HC + placebo, *Malassezia* spp. was detected in 18 out of 19 (95%) individuals, and in all 19 healthy rats (100%) that received FMT. There were no statistically significant differences in the prevalence of *Malassezia* between all groups (χ^2^ = 3.75, df = 5, *p* = 0.618).

*Cryptococcus neoformans* was detected in only one healthy rat before FMT and in one MAM-exposed rat after receiving FMT from a healthy donor.

*Methanobrevibacter smithii* and *Methanosphaera stadtmanae* were not detected in any of the rats (Table 2).

**Table 2:** The impact of FMT on detection rates (percentage of fecal samples positive for selected fungal and archaeal species) in rats prenatally exposed to MAM. Data are presented as % of positive samples per group, based on qPCR detection

Treatments	Positive fecal samples (%)				
	C. *tropicalis*	*Malassezia* spp.	C. *neoformans*	M. *stadtmanae*	M. *smithii*
MAM + (*n* = 48)	19%	100%	2%	0%	0%
HC (*n* = 39)	10%	100%	0%	0%	0%
MAM + placebo (*n* = 22)	95%	100%	0%	0%	0%
MAM + FMT (*n* = 24)	100%	96%	4%	0%	0%
HC + placebo (*n* = 19)	100%	95%	0%	0%	0%
HC + FMT (*n* = 19)	100%	100%	0%	0%	0%

FMT - fecal microbiota transplantation; HC - healthy control; MAM - methylazoxymethanol acetate

For each positive sample, the Cq value obtained during qPCR was used to determine the exact number of fungal cells (expressed as CFU per gram of stool), based on a standard curve generated from 10-fold serial dilutions of a reference strain with a known concentration.

The average CFU of *C. tropicalis* per gram of stool for the MAM-exposed group before FMT (in the 9 positive samples) was 8.49 × 10^1^, while for the HC group it was 5.84 × 10^1^. In the MAM + placebo group, the average CFU/g in positive samples increased to 1.55 × 10^3^. For MAM + FMT group, the average CFU/g was reduced to 6.90 × 10°. In HC + placebo, the average CFU/g was 2.07 × 10^3^. Finally, in rats from the HC + FMT group, the average CFU/g was 1.46 × 10^3^. Statistical analysis using Kruskal-Wallis revealed a significant difference between groups (H = 68.13; N_MAM_ = 48, N_HC_ = 39, N_MAM+placebo_ = 22, N_MAM+FMT_ = 24, N_HC+placebo_ = 19; *p* < 0.001).

The average CFU per gram of stool for *Malassezia* spp. in the MAM-exposed group before FMT was 1.15 × 10^6^, while in the HC group it was 1.43 × 10^6^. In the MAM + placebo group, the average CFU/g decreased to 5.26 × 10^5^, and in MAM + FMT group, it was 4.99 × 10^5^. Among rats from HC + placebo group, the average CFU/g was 7.75 × 10^5^, while healthy rats receiving FMT had an average CFU/g of 8.75 × 10^5^. Statistical analysis using the Kruskal-Wallis test revealed significant differences between groups (H = 42.11; N_MAM_ = 48, N_HC_ = 39, N_MAM+placebo_ = 22, N_MAM+FMT_ = 24, N_HC+placebo_ = 19; *p* < 0.001). *Malassezia* abundance was significantly higher before FMT in both MAM-exposed and healthy groups compared to their respective post-FMT placebo and FMT-treated subgroups). No significant differences were observed between the post-FMT placebo and FMT-treated subgroups within either MAM-exposed or healthy groups.

Due to the extremely low prevalence of *C. neoformans* in the studied groups, (in 2 samples) calculation of average CFU per gram of stool was not performed, as it would not provide meaningful or representative data. The quantitative assessment of selected fungal in positive fecal samples are summarized in Table 3.

**Table 3:** The abundance of selected fungal species (expressed as colony-forming units/g - CFU/g) in positive fecal samples from rats prenatally exposed to MAM and control groups (HC), depending on the treatment administered (FMT or placebo). Data are presented as mean value. Quantification was performed using qPCR assays with standard curves generated from reference strains

Treatment	Mean quantitative values in positive samples (CFU/g)	
	C. *tropicalis*	*Malassezia* spp.
MAM (*n* = 48)	8.49 × 10^1^	1.15 × 10^6^
HC (*n* = 39)	5.84 × 10^1^	1.43 × 10^6^
MAM + placebo (*n* = 22)	1.55 × 10^3^	5.26 × 10^5^
MAM + FMT (*n* = 24)	6.90 × 10°	4.99 × 10^5^
HC + placebo (*n* = 19)	2.07 × 10^3^	7.75 × 10^5^
HC + FMT (*n* = 19).	1.46 × 10^3^	8.75 × 10^5^

CFU - colony-forming units; FMT - fecal microbiota transplantation; HC - healthy control; MAM - methylazoxymethanol acetate

## Discussion

In this study, we investigated the presence and dynamics of selected fungal (*C.tropicalis*, *Malassezia* spp., *C. neoformans*) and archaeal species (*M. smithii*, *M. stadtmanae*) in the gut microbiome of MAM-exposed and healthy control rats, with a focus on the effects of fecal microbiota transplantation. Prior to FMT, *C. tropicalis* was more prevalent in MAM-exposed rats compared to controls, however, its colonization increased to nearly 100% in all groups fallowing FMT or placebo administration, suggesting that may be attributed to role of natural microbiome maturation rather than transplantation itself. *Malassezia* spp. were commonly detected before FMT but decreased after both FMT and placebo treatment, likely due to the administration procedure rather than specific microbial transfer. *C. neoformans* was only sporadically detected and methanogenic archaea were absent across all groups. These results suggest that FMT exerts limited effects on fungal and archaeal populations in young rats, and that the observed changes may be more closely related to host developmental processes and experimental conditions.

The fungal and archaeal species selected for this study were chosen based on their documented presence in the gastrointestinal tract and their potential involvement in host immune responses and gut-brain interactions [21, 33, 34]. Although *Malassezia* spp. are primarily known as skin commensals, recent studies have reported their occurrence in the gut microbiome, where they may influence mucosal immunity and contribute to inflammatory conditions [35, 36]. *C. tropicalis* and *C. neoformans* are opportunistic yeasts that can proliferate under dysbiotic conditions and are associated with systemic infections and altered immune function [37, 38]. In the context of neuropsychiatric disorders such as schizophrenia, fungal dysbiosis has been proposed as a potential contributing factor through immune-mediated or metabolic mechanisms [37, 39]. As for the archaeal targets, *M. smithii* is recognized as the most abundant archaea in the human and animal gut microbiota [40], playing a key role in methanogenesis by consuming hydrogen and contributing to microbial syntrophy [41]. *M. stadtmanae*, although less dominant, is notable for its strong immunostimulatory properties and has been linked to proinflammatory states [42], which may be relevant in the context of schizophrenia-associated gut-immune-brain dysregulation. The selection of these specific microorganisms provides insight into how their presence is shaped in schizophrenia-like rats and enables comparisons with existing research on microbial alterations in neuropsychiatric conditions.

While our study focused on the fungal and archaeal components of the gut microbiome in a rat model of schizophrenia, it is important to interpret these findings in the broader clinical and biological context of the disorder. Dysbiosis, including altered abundance of *Candida* or *Malassezia,* may contribute to schizophrenia through immune-mediated mechanisms, impaired intestinal barrier function, and interactions with the kynurenine pathway [16, 43]. Although direct correlations between specific microbial taxa and clinical symptoms remain to be fully established, studies like ours are essential for identifying potential links. Importantly, if the role of particular microorganisms in schizophrenia will be proven, this would open the possibility for targeted interventions, such as probiotic supplementation or fecal microbiota transplantation, which could offer novel therapeutic strategies [44]. These highlight the need for further studies to explore targeted microbiome-based interventions in schizophrenia. Translating such studies to human participants and clinical settings would allow for the assessment of whether modulation of gut microbiota composition or metabolic activity may help alleviate symptoms. Comprehensive integration of microbiological, immunological and metabolic data may contribute to the development of personalized therapeutic approaches in schizophrenia, potentially opening new alternatives for improving treatment outcomes in patients resistant to conventional treatments.

The protocol for our entire experiment was developed, among other factors, based on our previous analyses. They indicated that, for conducting therapeutic experiments using FMT, fecal samples from older rodents (35 and 42 days of age) seem to be the most valuable. In addition, a mixture of stool samples from both males and females seems to be the most effective in terms of the richness of bacterial and fungal composition.

Before FMT, we observed a slightly higher prevalence of *C. tropicalis* in MAM-exposed rats compared to healthy controls (Table 2) accompanied by a higher *C. tropicalis* abundance (Table 3). These findings are consistent with previous studies reporting increased levels and abundance of various *Candida* species in individuals with schizophrenia. Yuan et al. [45] identified an overrepresentation of the opportunistic pathogen *C. albicans*, along with a reduction in populations of beneficial fungi such as *Saccharomyces cerevisiae*, known for its immunomodulatory properties. Overgrowth of *Candida* has been associated with chronic inflammation and increased intestinal permeability, potentially contributing to neuroinflammatory processes through the production of pro-inflammatory molecules such as β-glucans. Importantly, a *Candida*-dominant enterotype was correlated with more severe psychotic symptoms and increased levels of systemic inflammation [37]. In a related study, Chinese scientists [46] demonstrated that among drug-naive, first-episode schizophrenia patients, the presence of a *Candida*-dominated gut profile was more common in individuals with a higher polygenic risk score (PRS) for schizophrenia. In turn, Sevarance et al. [33] have shown that males with schizophrenia have significantly higher levels of serum IgG antibodies against *C. albicans* compared to healthy controls, suggesting a possible role of fungal antigens in immune activation and disease pathology. Interestingly, probiotic interventions have been reported to normalize *Candida*-specific antibody levels and reduce gastrointestinal symptoms in individuals with schizophrenia, particularly in males [47]. Although these findings show that probiotics can be effective, it remains unclear whether their beneficial effects are due to actual changes in the gut fungal community or only from lowering the levels of fungal antigens in the blood. In contrast, our study using a more comprehensive method to improve the microbiome - fecal microbiota transplantation, did not show a clear effect on *C. tropicalis* populations. After FMT or placebo (physiological saline) administration, the presence of *C. tropicalis* increased to nearly 100% across all groups (Table 2), regardless of whether the rats received microbiota transplantation from healthy or MAM-exposed donors, or just the placebo. In our opinion, this rapid and abundant colonization after FMT/placebo treatment may not be directly related to the intervention itself but rather to the age of the animals. The rats were 42 days old prior to the start of FMT, and the effectiveness of the procedure was assessed 44 days later. This extended period of over six weeks could naturally contribute to the increased abundance of *C. tropicalis*, as gut fungal populations, including many yeasts, tend to increase with host maturation. Therefore, the observed rise in *C. tropicalis* colonization may reflect this natural developmental process rather than a direct effect of FMT. Increased presence of *C. tropicalis* post-FMT may also suggest the possibility of environmental contamination or cross-colonization between experimental groups especially considering that the administration of placebo resulted in similar fungal colonization levels as observed after FMT. Additionally, our findings may indicate that FMT has limited capacity to modulate fungal populations in the gut, particularly when donor material lacks sufficient fungal diversity or concentration.

Referring to the *Malassezia* results, we found that the presence of these fungi in the intestines of both MAM-exposed group and HC before FMT was ubiquitous (100% or nearly 100% in each group) confirming that *Malassezia* constitutes a natural component of the gut microbiota. Contrary to previous reports [48] we did not observe significant differences in *Malassezia* abundance between the MAM-exposed and HC groups (Table 3). Limited studies on these fungi in schizophrenia have reported higher levels of *Malassezia* but in the oral mycobiota of patients. Interestingly, this correlated with proinflammatory markers, suggesting a possible role in the pathophysiology of this disorder [48, 49]. However, it is important to note that our study was conducted on stool samples, whereas the above-mentioned studies analyzed tongue coat swabs. Moreover, we used a rat model with strictly controlled diet and environmental factors. In humans, *Malassezia* abundance and overall mycobiome composition are strongly influenced by diet, particularly fat intake, as well as other lifestyle factors [50–53], which are difficult to standardize. The controlled dietary conditions in animal models might therefore partly explain the lack of observed differences in *Malassezia* levels between the schizophrenia-like and control groups.

It should be noted that although the rats in our study were just over 6 weeks old at the start of FMT, corresponding approximately 4 human years in terms of sexual maturity, they had not yet reached social maturity, which in rats occurs around 7 months of age (equivalent to approximately 21 human years) [54]. The fecal samples in our study were collected when the rats were 86 days old, which can be compared to about 8.5 years in human age. This developmental stage is still early compared to most human studies on schizophrenia, which typically involve adult patients with chronic and long-standing disease. Additionally, early-onset schizophrenia during childhood or adolescence is extremely rare [55]and thus there is a lack of microbiome studies focused on children with schizophrenia to make direct comparisons. These age and developmental differences likely contribute to the discrepancies observed between our animal model and human clinical data regarding *Malassezia* abundance and its potential role in disease pathophysiology. Therefore, the increased abundance of *Malassezia* spp. often observed in individuals with various mental illnesses [49, 56, 57] may be a secondary consequence of long-term disease process, medication, lifestyle factors, or comorbid conditions, rather than a primary cause of the disorder.

Interestingly, we observed significantly higher average CFU values in both the MAM-exposed and HC groups before FMT compared to the levels after administration of placebo or FMT (Table 3). However, the lack of differences between the post-FMT and post-placebo subgroups indicates that the microbiota transplantation procedure itself, regardless of whether donor feces or saline was administered, was associated with a reduction in *Malassezia* spp. abundance in the gut. While we initially expected such an effect only after microbiota transplantation, the observation of a similar trend following placebo administration suggests that the procedure of administering the solution itself may have influenced the gut environment and *Malassezia* levels. This is especially relevant because, in our study, the solution was administered via a gastric tube, an invasive method that can induce short-term stress and physiological changes in the gastrointestinal tract. The mechanical action of the tube insertion and the administration of the solution may alter the gut microenvironment by affecting humidity, pH, and motility, which may transiently reduce *Malassezia* spp. colonization. Additionally, the procedure could induce mild immune responses or disrupt the microbial habitat, contributing to the observed decline. Therefore, the reduction in *Malassezia* abundance in both the placebo and FMT groups likely reflects the impact of the administration procedure and natural fluctuations in the gut mycobiome, rather than a specific effect of the microbiota transplant. Future studies should also consider including a no-intervention control group and exploring less invasive delivery methods to more clearly distinguish the specific effects of FMT on gut fungal communities.

*Cryptococcus neoformans* is a known opportunistic pathogen with neurotropic potential, occasionally associated with central nervous system infections and, in rare cases, neuropsychiatric manifestations such as psychosis [58, 59]. The fungus is capable of inducing neuroinflammation, modulating immune responses, and potentially interacting with neurotransmitter systems [60]. Despite these properties, there is currently no robust experimental or clinical evidence directly implicating *C. neoformans* in the pathogenesis of schizophrenia. Our findings are consistent with this lack of evidence. *C. neoformans* was not detected in any MAM-exposed rats prior to FMT, suggesting it does not naturally colonize the gut of rodents. Interestingly, *C. neoformans* was identified in one MAM-exposed rat after receiving FMT from a healthy donor. Considering that the same fecal material was used for all animals, the detection of *C. neoformans* in only one MAM-exposed rat after FMT is likely due to contamination during either the FMT administration or subsequent sample processing and qPCR analysis, rather than natural colonization. Our results support the notion that *C. neoformans*, while biologically capable of affecting brain function under certain conditions, is not a consistent or defining feature of the gut mycobiome in experimental schizophrenia.

Methanogenic archaea, such as *M. smithii* and *M. stadtmanae*, have been considered in a limited number of studies in the context of mental illnesses; however, due to their potential role in the gut-brain axis, as well as their impact on intestinal gas metabolism and the immune system, they are receiving growing scientific interest [61–63]. These methanogens may not only impact inflammation but also modulate gut microbiota and influence cognitive function through complex interactions within the microbial ecosystem. For example, Fumagalli et al. [23] demonstrated that *M. smithii* abundance positively correlated with cognitive performance, along with distinct bacterial profiles and enriched metabolic pathways related to energy, butyrate, and bile acid metabolism. In turn, Misiak et al. [22] demonstrated an increased abundance of *Methanobrevibacter* in individuals with chronic schizophrenia, which correlated with elevated levels of immune markers, including IL-6, IFN-γ, RANTES, and IP-10, as well as with an altered lipid profile, notably reduced HDL levels. These findings suggest a potential involvement of methanogenic archaea in the pathophysiology of schizophrenia, possibly through immunometabolic mechanisms linked to host-microbiota interactions. Nevertheless, in our study, we did not detect the presence of *M. smithii* or *M. stadtmanae* in any fecal samples from rats, regardless of whether they belonged to the HC group or the MAM-exposed group. This may be related to the age of the rodents - young rats may have a less developed and less diverse gut microbiota, and the abundance of methanogens increases with age [64, 65]. Alternatively, it is possible that these particular archaea are not commonly present in rats or exist at very low level, making their detection by qPCR challenging. The literature lacks unequivocal reports confirming the consistent presence of *M. smithii* and *M. stadtmanae* in rats, indicating the need for further research in this area. Notably, in the aforementioned study by Misiak et al. [22], increased abundance of *Methanobrevibacter* was observed in patients with chronic schizophrenia. In contrast, our model represents an early phase of the disorder, using young rats corresponding to the period of human childhood. It is possible that archaea emerge only with the progression of the disease - we have observed this pattern in our previous study on IBD patients [66].

In summary, our study evaluated the impact of fecal microbiota transplantation on selected gut fungi and archaea in MAM-exposed rats - a neurodevelopmental model of schizophrenia. *Candida tropicalis* was slightly more prevalent in MAM-exposed rats prior FMT; however, after administration of FMT or placebo, its colonization increased to nearly 100% across all groups, suggesting these changes are due to natural microbiome development rather than the transplantation itself. *Malassezia* spp. were commonly detected, and their abundance decreased following both FMT and placebo treatment, likely as a result of the invasive procedure used to administer the solution. *Cryptococcus neoformans* and methanogenic archaea were not identified. The results indicate that FMT has a limited effect on the fungal component of the gut microbiome in young rats, and observed changes may reflect natural processes of microbiome maturation or the influence of the procedures used. Our findings, including the absence of selected archaeal taxa, highlight the need for further research into the microbiome’s role in schizophrenia.

## Data Availability

The datasets generated during and/or analysed during the current study are available from the authors upon reasonable request.

## Abbreviations

CFU: Colony forming unit
FMT: Fecal microbiota transplantation
GD17: 17^th^ gestation day
GWAS: Genome-wide association studies
HC: Healthy control
HDL: High-density lipoprotein
HPA: Hypothalamic-pituitary-adrenal axis
IBD: Inflammatory bowel disease
IDO: Indoleamine 2,3-dioxygenaseIFN-γ - interferon gamma
IL-6: Interleukin 6
IP-10: Interferon gamma-induced protein 10
MAM: Methylazoxymethanol acetate
NMDARs: N-methyl-D-aspartate receptors
TDO: Tryptophan 2,3-dioxygenase
qPCR: Quantitative polymerase chain reaction
PND21: 21^st^ postnatal day
PRS: Polygenic risk score
RANTES: Regulated upon activation, normal T cell expressed and secreted
αnAChR: α7 nicotinic acetylcholine receptors
